# Zero-heat flux skin temperature tracks nasopharyngeal temperature during cardiac surgery with cardiopulmonary bypass: a prospective observational study

**DOI:** 10.1186/s12871-026-04068-4

**Published:** 2026-07-14

**Authors:** Kyoung-Woon Joung, Chung-Sik Oh, Ju-Youn Kim, Suryeon Lee, Dong-Kyu Lee, Tae-Yop Kim, In-Cheol Choi

**Affiliations:** 1 Department of Anesthesiology and Pain Medicine, Asan Medical Center, University of Ulsan College of Medicine, Seoul, Korea; 2 Laboratory for Cardiovascular Dynamics, Asan Medical Center, University of Ulsan College of Medicine, Seoul, Korea; 3 Department of Anesthesiology and Pain Medicine, Konkuk University Medical Center, Konkuk University School of Medicine, 120-1, Neungdong-Ro, Gwangjin-Gu, Seoul, 05030 Korea; 4 Institution for Patient-Centered Goal-Directed Strategy, Konkuk University, Chungju, Korea; 5 Department of Anesthesiology and Pain Medicine, Uijeongbu Eulji University Medical Center, Uijeongbu, Korea; 6 Department of Anesthesiology and Pain Medicine, Dongguk University Ilsan Hospital, Goyang, Korea

**Keywords:** Cardiac surgery, Cardiopulmonary bypass, Skin Temperature, Zero-heat flux

## Abstract

**Background:**

Monitoring both nasopharyngeal temperature (T_NP_) and arterial blood temperature at the CPB arterial outlet is routinely used in cardiac surgery with cardiopulmonary bypass (CPB). We examined the agreement and temperature tracking ability of zero-heat-flux skin temperature (T_ZHF_) compared with T_NP_ in cardiac surgery.

**Methods:**

Of 40 patients scheduled to undergo cardiac surgery with CPB, paired T_ZHF_ and T_NP_ measurements were recorded for 33 patients, yielding 8610 measurements across pre-CPB, CPB-cooling, CPB-maintenance, CPB-rewarming, and post-CPB.

**Results:**

In the final, trimmed dataset, T_ZHF_ and T_NP_ at 30 min after CPB weaning, the primary endpoint, showed similar mean values (35.57 ± 0.69 °C vs. 35.69 ± 0.61 °C; *P* = 0.181), with a mean difference of –0.12 °C (95% Confidence Interval: –0.29 to 0.06). Bland–Altman analysis showed a small mean bias between T_ZHF_ and T_NP_ across all intraoperative phases, ranging from − 0.55 to 0.28 °C. However, the limits of agreement (LOA) exceeded the clinically acceptable ± 0.5 °C during CPB-cooling and CPB-rewarming. Mixed-effects modeling showed considerable between-patient variability in the difference between T_ZHF_ and T_NP_ measurements during periods of rapid temperature change. Consistent with the Bland–Altman results, correlation coefficients between T_ZHF_ and T_NP_ measurements remained high (ranging from 0.798 to 0.958), and their intraclass correlation coefficients were high (ranging from 0.82 to 0.99) across all intraoperative phases.

**Conclusions:**

Our results indicate that T_ZHF_ tracked T_NP_ trends, but its large LOA limits its accuracy and reliability. Simultaneous blood temperature monitoring would enhance T_ZHF_’s effectiveness, as is currently done in cardiac surgery, where both blood temperature and T_NP_ are monitored.

**Trial registration:**

ClinicalTrials.gov Identifier: NCT04160845.

**Supplementary Information:**

The online version contains supplementary material available at https://doi.org/10.1186/s12871-026-04068-4.

## Introduction

Precise temperature monitoring is essential during cardiac surgery with hypothermic cardiopulmonary bypass (CPB). While hyperthermia poses the risks of prolonged neurophysiological and renal impairment, cerebral hyperthermia is detrimental and associated with negative outcomes after cardiac surgery [1–4]. At overt stroke, hyperthermia during CPB rewarming exacerbates the extent of tissue injury, and postoperative hyperthermia correlates with a greater degree of cognitive dysfunction measured after cardiac surgery [5]. Therefore, temperature management has focused on preventing excessive blood heating and postoperative cerebral hyperthermia during the CPB rewarming phase.

While the nasopharynx, oropharynx, esophagus, axilla, bladder, rectum, and pulmonary artery catheter (PAC) can be used as sites for surrogate blood or core temperature measurements, nasopharyngeal (NP) temperature (T_NP_) has been preferred in cardiac surgery employing hypothermic CPB [5]. Due to the delay in redistributing heat and reaching thermal equilibrium between tissue and blood, T_NP_ did not reflect well with blood and brain temperatures, especially during rapid temperature changes [6, 7]. Therefore, T_NP_ monitoring is routinely used alongside blood temperature monitoring at the arterial outlet of the CPB oxygenator during cardiac surgery [8]. From a clinical perspective, blood temperature measured at the arterial CPB outlet may be the most reliable estimate of brain temperature [5]. However, it also cannot accurately estimate brain temperature during rapid temperature changes [5, 9, 10].

Skin temperature monitoring employing the zero-heat-flux technique (T_ZHF_) has been introduced as a reliable, non-invasive, and seamless method for perioperative monitoring core temperature [11, 12]. T_ZHF_ agreed well with other modalities, such as T_NP_ and T_PAC_, in normothermic and mild hypothermic temperature ranges (> 32 ℃) during cardiac surgery [13–15]. Meanwhile, similar to T_NP_ monitoring, T_ZHF_ requires a time lag for establishing temperature equilibrium (the isothermal tunnel between the skin and deeper tissues), which can compromise its accuracy and reliability, especially during CPB cooling and rewarming phases [16, 17].

There is no standard method to measure both blood and brain temperatures. Therefore, our study compared the two methods, T_ZHF_ and T_NP_, rather than validating one against the other. The authors assumed that if T_ZHF_ performs similarly to T_NP_ and its changes align well with those of T_NP_, T_ZHF_ could be considered as a clinically acceptable alternative to T_NP,_ which is routinely used in combination with blood temperature monitoring for cardiac surgery.

Therefore, we compared T_ZHF_ and T_NP_ measurements during the post-CPB period, as the primary measure, in patients undergoing cardiac surgery with hypothermic CPB.

As secondary measures, we examined agreements and correlations between changes in T_ZHF_ and T_NP_ throughout the intraoperative period in these patients. We also analyzed intra-individual clustering and time-dependent effects of their repeated measurements.

## Methods

### Study population, inclusion, and exclusion criteria

This prospective observational study was performed in a tertiary university hospital from March 2021 to April 2022. The research protocol was approved by the Institutional Review Board (AMC IRB 2021–0073) and registered at ClinicalTrials.gov (NCT04160845).

Patients scheduled for elective cardiac surgery were enrolled, and written informed consent was obtained from all subjects at enrollment. Exclusion criteria included not undergoing CPB and patient withdrawal, even after surgery. Temperature data due to the exposure of a nasopharyngeal thermistor (detected by the inadvertent withdrawal of the NP thermistor and the gap > 3 °C) during an extended period (longer than 15 min), or had changed surgical plans during surgery.

### Anesthesia management

Perioperative management strategies for the present study were described in a previous study [18]. General anesthesia was induced and maintained with sevoflurane-remifentanil-rocuronium or propofol-remifentanil-rocuronium. Before induction of anesthesia, electrocardiogram, near-infrared regional cerebral oxygen saturation, invasive blood pressure, and heart rate were monitored. After anesthesia induction and endotracheal intubation, a pulmonary artery catheter and a probe of transesophageal echocardiography were placed. Before and after the CPB application, O_2_/air mixture (FiO_2_ 0.3–0.5) was ventilated with a tidal volume of 6 ml/kg, a respiratory rate for maintaining the end-tidal CO_2_ of 35–40 mmHg, and a positive end-tidal pressure of 6–8 mmHg. For cardiac surgery under the moderate hypothermic CPB, heparin 4–5 mg/kg was administered, and the patient underwent systemic cooling using CPB. A bloodless cardioplegia was administered for cardiac arrest during CPB. Protamine 2–3 mg/kg was administered to neutralize the heparin effect at the end of CPB. After the end of surgery, patients were transferred to the intensive care unit (ICU) under tracheal intubation and remained sedated with dexmedetomidine till extubation.

### Temperature monitoring and management

Before anesthesia induction, a skin temperature sensor (3 M ™ SpotOn™ temperature monitoring system, 3 M, Canada) was placed on the forehead skin surface according to the manufacturer's recommendations to monitor T_ZHF_. Immediately after anesthesia induction, two additional types of thermometers were placed for monitoring T_NP_ and T_PAC_: a NP thermistor (Top Probe™, esophageal stethoscope temperature sensor, Meditop company, Korea) was inserted into the patient’s nose and placed at a depth equal to the distance between the nares and the earlobe [19]; and PAC (CCOmbo Swan-Ganz advanced technology pulmonary artery catheter™, Edwards Lifesciences, USA) was placed in the right internal jugular vein.

Adequate positioning and performance of all three thermistors were confirmed at intervals throughout the study, except during the PAC withdrawal period in the surgical procedure under CPB.

To maintain normothermia during pre-CPB and post-CPB periods, a forced-air warming blanket (3 M™ Bair Hugger™ system, 3 M, Canada) and a cooling-heating blanket underneath the patient (Ethicon Megadyne™, dual cord patient return electrode pad, Megadyne Medical Products INC, USA, and Blanketrol® III, Cincinnati Sub-Zero, USA) were applied. The ambient temperature in the operating room was maintained at approximately 20℃ during the surgery.

Direct blood cooling and warming using the CPB pump were applied for systemic cooling to achieve hypothermic CPB and CPB rewarming. Especially for CPB rewarming, direct blood warming was initiated to achieve a target T_NP_ of 36.0 ℃, and the difference between the blood temperature in the CPB arterial outlet and T_NP_ was kept from exceeding 2.0 °C. At the later part of CPB rewarming, the blood temperature at the CPB arterial outlet was kept from exceeding 37 °C. Indirect skin cooling and warming were applied by using a blanket underneath the patient during the entire procedure.

### Data acquisition

We utilized an electronic data acquisition system (Vital Recorder program version 1.7.4, Department of Anesthesiology and Pain Medicine, Seoul National University College of Medicine, Korea) and a laptop computer connected to multiple patient monitoring devices via serial cables to electronically record all measured temperature data, including T_ZHF_, T_NP,_ and T_PAC_ at 1-min intervals throughout the study.

We divided the entire intraoperative period into five phases: the phase before initiating CPB (Pre-CPB), the phase of systemic cooling under CPB (CPB-cooling), the phase of maintaining hypothermic CPB (CPB-maintenance), the phase of systemic rewarming under CPB (CPB-rewarming), and the phase after weaning from the CPB (Post-CPB). CPB-cooling was defined as the time from the beginning of the T_ZHF_ drop to the time at which the lowest level was achieved: the temperature gradient between the heat exchanger and the arterial inflow must not exceed 10 ℃ during CPB cooling [8]. CPB-rewarming was defined as the time from the start of the T_ZHF_ rise to the time at which 35℃ was reached. The temperature gradient between the blood and arterial outlet was kept within 2 °C during CPB rewarming. Then, we categorized the collected data of T_ZHF_, T_NP_, and T_PAC_ measurements for phase-specific analyses.

T_PAC_ data during CPB-cooling, CPB-maintenance, and CPB-rewarming were excluded from the analysis due to irregular and inconsistent measurements observed across these phases. Temperature data lower than 25℃ during CPB were excluded from the analyses.

Before statistical analysis, data preprocessing was performed: all temperature data were carefully examined, and data points with abrupt extreme temperature changes were manually identified and excluded from the dataset.

### Primary and secondary measures

The primary measure of our study is to analyze the agreement between T_ZHF_ and T_NP_ measurements 30 min after the CPB weaning. We defined the clinically acceptable agreement (i.e., 95% limits of agreement, LOA) between T_ZHF_, T_NP_, and T_PAC_ measurements within 0.5℃ [20–22].

As secondary measures, we evaluated the phase-specific analyses of T_ZHF_, T_NP_, and T_PAC_ measurements, as well as the correlations and agreements between T_ZHF_ and T_NP_ measurements. Additional analyses were also performed to determine the consistency of the agreement within subjects, time-dependent effects on the consistency across multiple repeated measurements within subjects, and site-to-site consistency during the periods of rapid temperature change.

### Statistical analysis

All statistical analyses were performed using R software (version 4.4.2, R Foundation for Statistical Computing, Vienna, Austria). A two-sided p-value of less than 0.05 was considered statistically significant throughout the study. Continuous data are presented as the mean ± standard deviation (SD) or median (25–75%). Missing values were addressed using a complete-case approach for each statistical procedure, without imputation.

The sample size was calculated for the primary comparison of paired T_ZHF_ and T_NP_ measurements 30 min after weaning from cardiopulmonary bypass. In the pilot data, the mean temperatures were 35.7 ± 0.55 °C for T_ZHF_ and 35.8 ± 0.40 °C for T_NP_, and the standard deviation of paired T_ZHF_ – T_NP_ differences was 0.46 °C. The clinically relevant mean difference was set at 0.25 °C. This value corresponded to half of the predefined clinically acceptable agreement margin of ± 0.5 °C and was chosen conservatively because the final primary endpoint involved comparison among three temperature measurements, including T_PAC_. By setting the detectable systematic difference to half of the acceptable margin, we aimed to ensure that potential pairwise deviations among the three measurements would remain within the clinically acceptable range. Using a paired t-test with a two-sided α of 0.05 and 80% power, 29 paired observations were required. Assuming a 10% dropout rate, the target sample size was increased to 33 patients.

For the primary measure, a paired t-test was performed to compare T_ZHF_ and T_NP_ at 30 min after weaning from CPB. Additionally, a repeated-measures analysis of variance was performed to compare T_ZHF_, T_NP_, and T_PAC_ measured 30 min after weaning from CPB. Mauchly’s test was used to evaluate the sphericity assumption of the within-subject factor. If sphericity was violated, Greenhouse–Geisser or Huynh–Feldt corrections were applied to adjust degrees of freedom and P-values. Post hoc pairwise comparisons were performed using a paired t-test with Holm adjustment.

To account for the significant overall differences, pairwise comparisons were conducted using paired t-tests with the Holm correction to adjust for multiple comparisons.

Agreement was evaluated using Bland–Altman analysis, through the mean difference, LOA, and corresponding 95% confidence intervals (CIs). Additionally, Pearson’s correlation coefficients were estimated to quantify the linear relationship between measured temperatures.

Additional complementary methods were employed to assess agreement among the measured temperatures. Intraclass correlation coefficients (ICC) were computed using a two-way mixed-effects model (ICC3) to assess the consistency of specific temperature-monitoring devices across repeated measurements within subjects. To account for time-dependent effects during rapid temperature changes (CPB-cooling and CPB-rewarming), residual values were derived from linear regression models that adjusted for time.

To quantify site-to-site consistency and to account for the time-dependent effects between T_ZHF_ and T_NP_ during rapidly changing temperatures (CPB-cooling and CPB-rewarming), residual-based intraclass correlation coefficients (Residual-ICC3) were then estimated using these residuals, under a two-way mixed-effects model assuming fixed measurement devices.

Time-dependent patterns of the measured temperatures were explored using raw and normalized time axes. Time-series mean plots were used to visualize the trends of T_NP_ and T_ZHF_ across CPB-cooling and CPB-rewarming. To examine the evolution of the temperature difference, bias-over-time analysis was performed using LOESS smoothing, which allows for the detection of non-linear trends in temperature discrepancies throughout each phase. In addition to time-dependent effects, a normalized progress scale was applied to each patient to account for individual variability in cooling and warming rates during CPB cooling and CPB rewarming. The relative progress of the cooling or warming process was expressed as a percentage. Using these standardized scales, the trend in temperature difference was analyzed to compare patterns across patients with varying target temperatures and durations.

To mitigate the variation in temperature changes between T_ZHF_ and T_NP_ (in terms of the gap between baseline and target temperatures and the duration of temperature change for CPB-cooling or CPB-rewarming), we adjusted the progression of temperature changes by normalizing the temperature difference and duration in each case.

Finally, since temperature monitoring generates repeated measurements over time, mixed-effects models were used to account for within-patient clustering. Each patient was considered as a random effect with both random intercepts and slopes during CPB-cooling and CPB-rewarming. Normalized durations of CPB-cooling or CPB-rewarming and the temperature gap (from the baseline temperature to the target temperature) during these rapid temperature changes were treated as fixed effects. Model fit and performance were evaluated using the Akaike information criteria, Bayesian information criteria, and log-likelihood values. Model diagnostics were also performed, including the examination of residuals through a histogram and normality assessment. The estimated random effects were visualized, and correlations between random intercepts and slopes were reported. The fixed and random effects were also visualized with predictive marginal plots to compare population-level effect with individual patient-specific trends.

Statistical analysis and visualization were performed using the following R packages: psych, ez, lme4, lmerTest, ggplot2, ggeffects, and lattice [23–29].

## Results

**Fig. 1:**
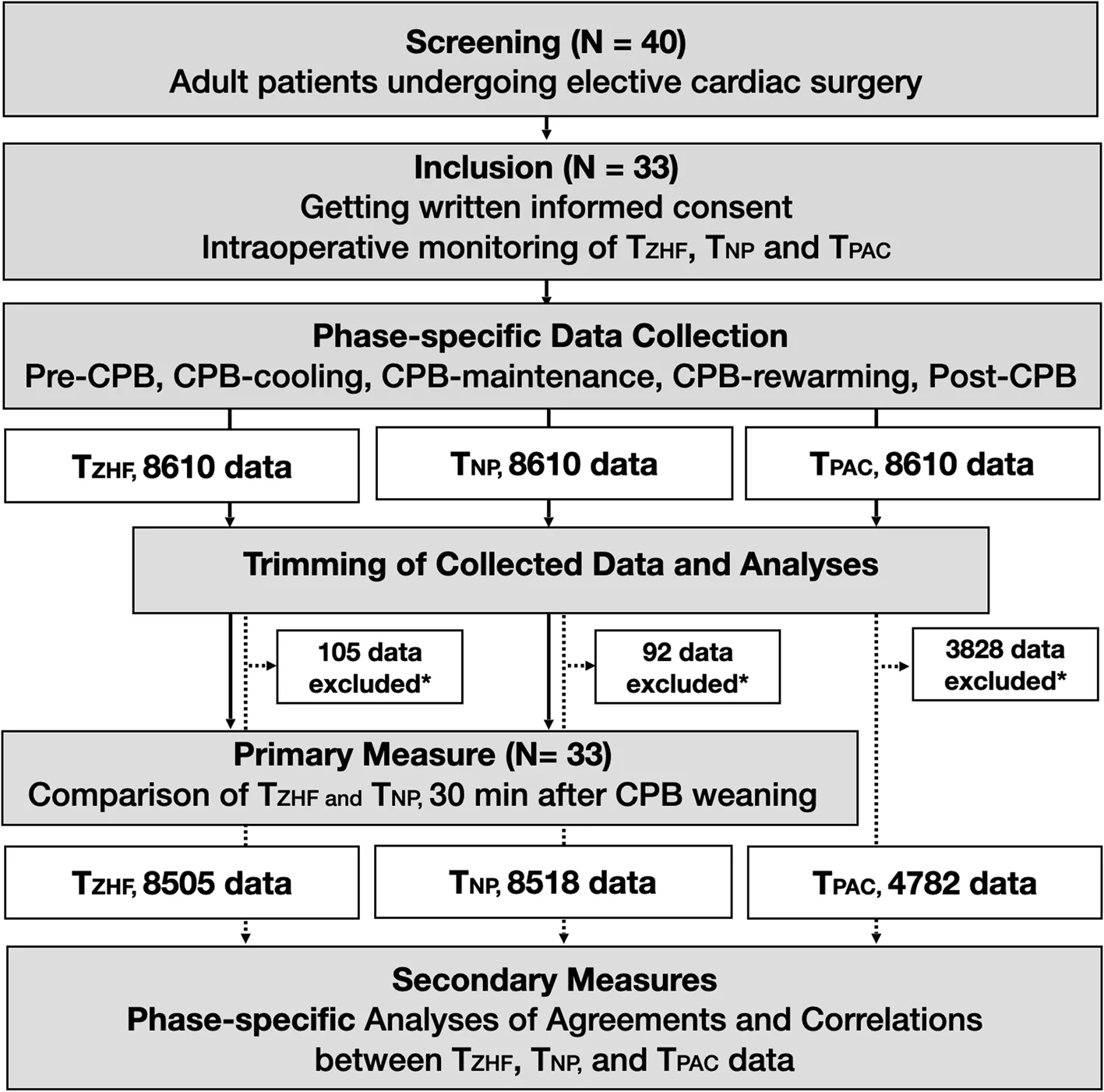
Study flow diagram. Abbreviations: T_ZHF_ = temperatures measured by ZHF skin temperature sensor, T_NP_ = temperatures measured by nasopharyngeal thermometer, T_PAC_ = temperature measured by PA catheter. CPB = cardiopulmonary bypass, Pre-CPB = phase before applying CPB, CPB-cooling = phase during the CPB cooling, CPB-maintenance = phase during the maintenance of hypothermic CPB (> 32 ℃), CPB-warming = phase during the CPB rewarming, Post-CPB = phase after weaning from CPB. * = T_ZHF_ and T_NP_ data lower than 25℃ were excluded from the analyses, and T_PAC_ data during CPB-cooling, CPB-maintenance, and CPB-rewarming were excluded from the analyses. The excluded data were regarded as missing values

Among 40 screened patients scheduled to undergo elective cardiac surgery, paired T_ZHF_ and T_NP_ measurements were recorded in 33 patients, yielding 8610 data sets of T_ZHF_, T_NP_, and T_PAC_ measurements across pre-CPB, CPB-cooling, CPB-maintenance, CPB-rewarming, and post-CPB (Fig. 1). Demographic and surgical data for the participating patients are presented in Table 1.

**Table 1:** Demographic data

Variables	
Age, yr	62.0 [53.0 ‒ 73.0]
Sex, female	20 (60%)
Height, cm	162.3 [156.0 ‒ 165.9]
Weight, kg	59.3 (53.9 ‒ 64.7)
Types of surgery	
Isolated AV surgery (AVR)	10 (30.3%)
Combined valve (AV and MV) surgery	6 (18.2%)
AV and aortic root surgery	5 (15.2%)
Other cardiac/aortic procedure	6 (18.2%)
Operation duration, min	260.0 [221.0 ‒ 369.0]
Duration of CPB, min	120.0 [91.0 ‒ 146.0]

Data are presented as numbers (%), or median [IQR]

*Abbreviations*: *AV* Aortic valve, *AVR* AV replacement, *CPB* Cardiopulmonary bypass, *MV* Mitral valve, *MVR* MV replacement, *MVP* MV plasty, *TAP* Tricuspid annuloplasty

The trimmed data, 105 in T_ZHF_, 92 in T_NP_, and 3828 in T_PAC_, were treated as missing or trimmed values and excluded from the analyses. The median length of temperature measurement (IQR) in each patient was 572 min (296‒1166 min). Trimming of data was performed to exclude T_ZHF_ and T_NP_ data lower than 25℃ and T_PAC_ data during CPB-cooling, CPB-maintenance, and CPB-rewarming. After trimming data, median observation times (IQR) of the pre-CPB, CPB-cooling, CPB-maintenance, CPB-rewarming, and post-CPB phases were 49.0 (IQR 36.0 ‒ 64.0) min, 81.5 (IQR 63.2 ‒ 100.0) min, 140.0 (IQR 119.0 ‒ 148.0) min, 148.0 (IQR 123.0 ‒ 196.0) min, and 261.0 (IQR 233.0 ‒ 365.0) min, respectively. The phase-specific summary of temperature measurements is presented in Table 2.

**Table 2:** Summary of measured temperature data during the 5 different intraoperative phases

Phase	T_ZHF_	T_NP_	T_PAC_
Pre-CPB	35.7 ± 0.7 (1540)	35.7 ± 0.6 (1525)	35.8 ± 0.6 (1472)
CPB-cooling	33.0 ± 2.1 (1247)	32.7 ± 2.4 (1248)	not included*
CPB-maintenance	31.7 ± 2.2 (694)	31.3 ± 3.1 (759)	not included*
CPB-rewarming	33.4 ± 2.3 (1493)	33.9 ± 2.1 (1517)	not included*
Post-CPB	35.6 ± 0.8 (3531)	35.7 ± 0.7 (3469)	35.9 ± 0.7 (3308)

Data are presented as mean ± SD of temperatures, ℃, (number of measurements)

*Abbreviations*: *T*_*ZHF*_ Temperatures measured by ZHF skin temperature sensor, *T*_*NP*_ Temperatures measured by nasopharyngeal thermometer, *T*_*PAC*_ Temperature measured by PA catheter, *Pre-CPB* Phase before applying CPB, *CPB-cooling* Phase during the CPB cooling, *CPB-maintenance* Phase during the maintenance of hypothermic CPB (> 32 ℃), *CPB-warming* Phase during the CPB rewarming, *Post-CPB* Phase after weaning from CPB

^*^PA catheter was withdrawn during the CPB

### Comparisons of T_ZHF_, T_NP_, and T_PAC_ measurements at 30 min after CPB weaning

**Fig. 2:**
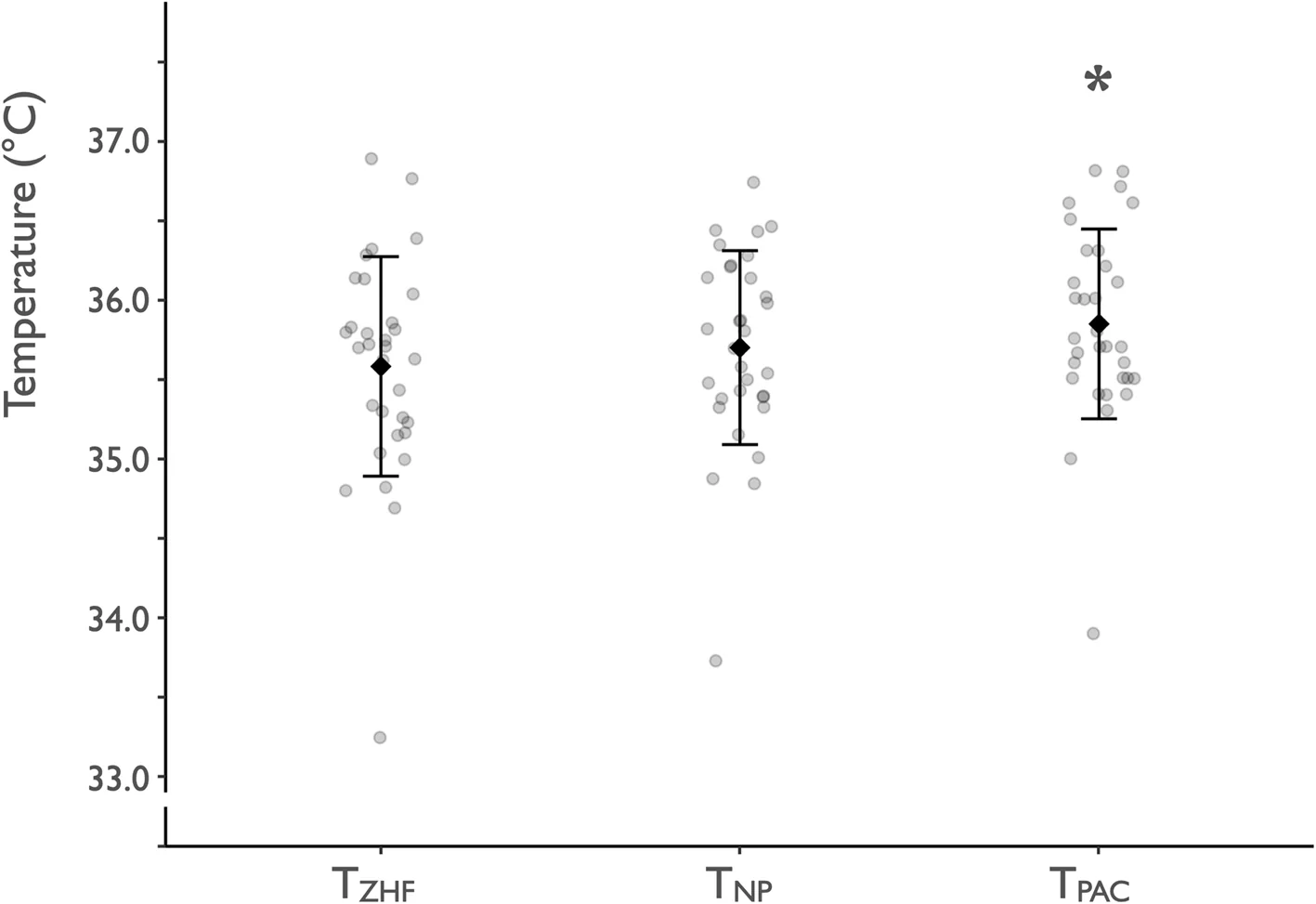
Comparisons of T_ZHF_, T_NP_, and T_PAC_ at 30 min after CPB weaning. Repeated measures ANOVA and post-hoc pairwise comparison using paired *t*-test with Holm correction. * = P < 0.05 vs. T_ZHF_. The primary measure was the comparison of T_ZHF_ and T_NP_ measurements 30 min after CPB weaning. Abbreviations: T_ZHF_ = temperatures measured by ZHF skin temperature sensor, T_NP_ = temperatures measured by nasopharyngeal thermometer, T_PAC_ = temperature measured by PA catheter

As the primary measure of the present study (Fig. 2), the measurements (mean ± SD) of T_ZHF_ of T_NP_ were not significantly different (35.57 ± 0.69 ℃ and 35.69 ± 0.61 ℃, respectively), and the mean difference of these two measurements was −0.12 ℃ (95% CI, −0.29 ‒ 0.06 ℃) (P = 0.181, Cohen’s *D* = 0.242). When compared with T_PAC_ (35.84 ± 0.59 °C), T_ZHF_ showed a slightly lower mean value, resulting in a mean difference of − 0.27 °C (95% CI − 0.45 to − 0.09 °C; pairwise comparison P = 0.014). In contrast, TNP and TPAC measurements were not significantly different at this time point (mean difference − 0.11 °C; pairwise comparison P = 0.181; overall repeated-measures ANOVA P = 0.008, generalized η^2^ = 0.030, partial η^2^ = 0.156).

### Agreement between T_ZHF_ and T_NP_ across intraoperative phases

**Fig. 3:**
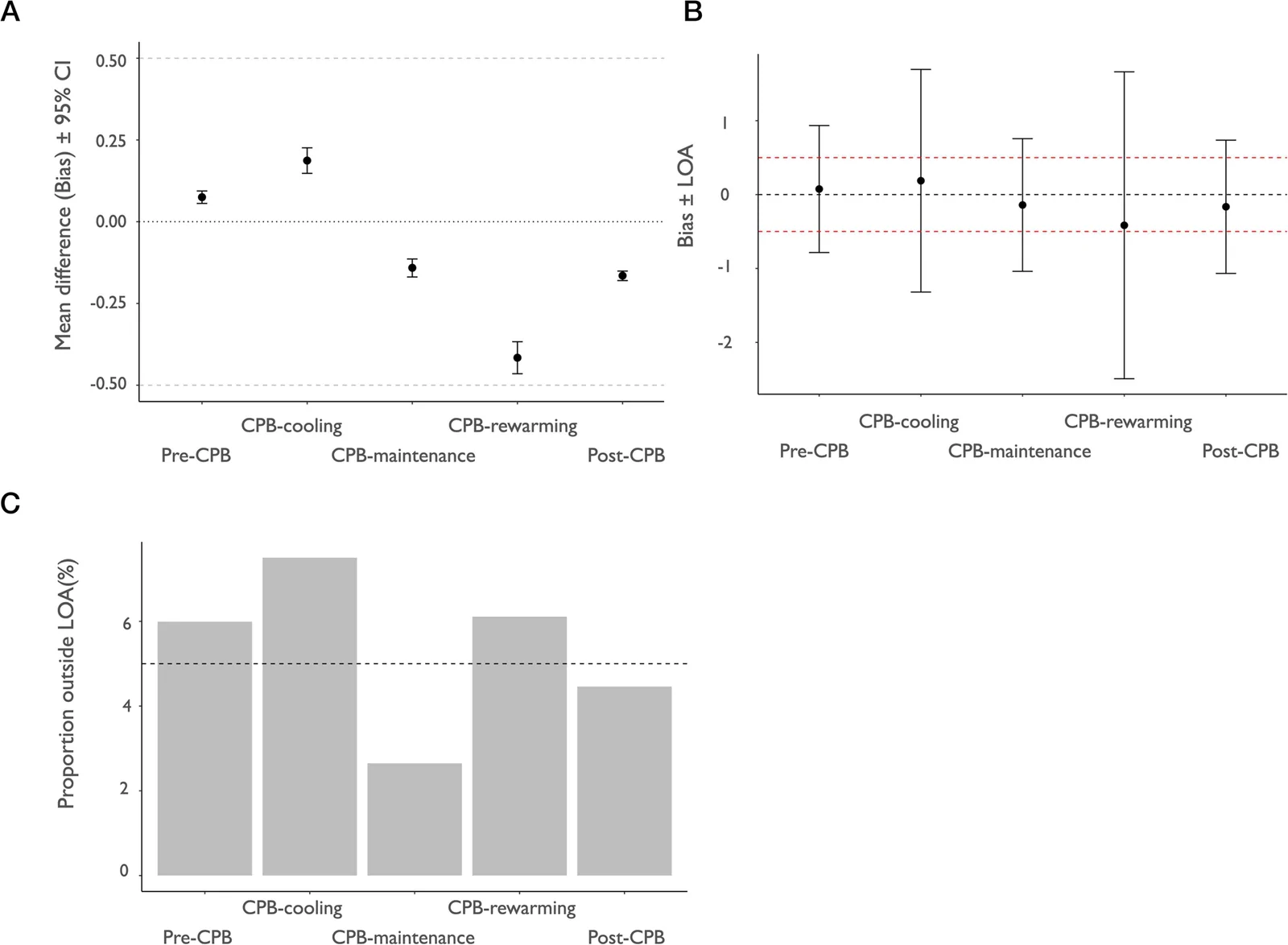
Agreement between T_ZHF_ and T_NP_ measurements during 5 different intraoperative phases**,** estimates from Bland–Altman analysis. **A** Mean difference and 95% CIs, **B** Bias and limit of agreement (± 0.5 ℃), C = Proportions outside the LOA. The dotted lines in B indicate the clinical margin, and the dotted line in C indicates the overall proportions of data outside the LOA. Abbreviations: T_ZHF_ = temperatures measured by ZHF skin temperature sensor, T_NP_ = temperatures measured by nasopharyngeal thermometer, 95% CI = 95% confidence interval, LOA = limit of agreement

Bland–Altman analysis demonstrated a small mean bias between T_ZHF_ and T_NP_ across all intraoperative phases, ranging from − 0.55 to 0.28 °C (Fig. 3, Supplementary Fig. 1, and Supplementary Table 1) However, the limits of agreement widened during phases involving rapid temperature changes. The LOA exceeded the predefined clinical margin of ± 0.5 °C in all phases, with particularly large values during CPB cooling and rewarming, reaching up to ± 2.3 °C.

The proportions of measurements exceeding the clinical margin of ± 0.5 °C were 6.7% during Pre-CPB, 5.1% during CPB-cooling, 0.9% during CPB-maintenance, 6.2% during CPB-rewarming, and 4.5% during Post-CPB.

Similarly, the agreements between T_ZHF_ and T_PAC_, as well as between T_NP_ and T_PAC_, showed that LOA exceeded the predefined clinical margin during the pre-CPB and post-CPB phases (Supplementary Table 1, Supplementary Fig. 2, and Supplementary Fig. 3).

### Temperature dynamics during CPB cooling and rewarming

During active temperature-modulation phases, T_ZHF_ exhibited a slower rate of temperature change than T_NP_ (Supplementary Fig. 4).

During CPB cooling, the difference between T_ZHF_ and T_NP_ increased during the early phase of cooling, peaking in the first quarter of the cooling process, and gradually decreased as the temperature approached the nadir (target temperature for rewarming and cooling).

During CPB rewarming, the difference between T_ZHF_ and T_NP_ was minimal at the beginning of rewarming. Subsequently, T_ZHF_ increased relative to T_NP_, resulting in a temperature difference of approximately 0.53 °C at the completion of rewarming.

### Inter-individual variability in temperature differences

Mixed-effects modeling demonstrated considerable variability between patients in the difference between T_ZHF_ and T_NP_ measurements during periods of rapid temperature change.

During CPB cooling, the overall population trend did not show a significant change in the temperature difference between devices (P = 0.204). However, substantial inter-individual variability was observed in the baseline difference (random intercept standard deviation = 0.44 °C).

During CPB rewarming, T_ZHF_ measurements were initially higher than T_NP_ (population-level intercept difference 0.39 °C, 95% CI 0.18–0.61 °C; P < 0.001), while the difference remained stable throughout the rewarming process (slope P = 0.233) (Supplementary Fig. 5, Supplementary Table 2).

These findings suggest that although T_ZHF_ closely followed the overall temperature trajectory observed with T_NP_, the magnitude of the difference between the devices varied across individual patients.

### Supporting consistency analyses

Consistent with the Bland–Altman results (Table 3, Supplementary Fig. 1), correlation coefficients between T_ZHF_ and T_NP_ remained high across all intraoperative phases, ranging from 0.798 to 0.958. Intraclass correlation coefficients (ICC3) also indicated strong consistency between the two monitoring methods; 0.86 (95% CI 0.84–0.87) at Pre-CPB, 0.95 (95% CI 0.95–0.96) at CPB-cooling, 0.99 (95% CI 0.99–0.99) at CPB-maintenance, 0.92 (95% CI 0.91–0.93) at CPB-rewarming, and 0.82 (95% CI 0.81–0.83) at Post-CPB. After adjusting for the rate of temperature change during CPB cooling and rewarming, the residual ICC values remained high (0.97 and 0.93, respectively), indicating consistent temporal trends between the two methods.

**Table 3:** Correlation and intraclass correlation coefficients between temperature measurements during 5 different intraoperative phases

Phases	T_ZHF_ vs. T_NP_			T_ZHF_ vs. T_PAC_
	Correlation	ICC3	Residual-ICC3	Correlation
Pre-CPB	0.867	0.86 (0.84–0.87)	*not performed*	0.864
CPB-cooling	0.959	0.95 (0.95‒0.96)	0.97 (0.96‒0.97)	not performed*
CPB-maintenance	0.798	0.99 (0.99‒0.99)	*not performed*	not performed*
CPB-rewarming	0.918	0.92 (0.91‒0.93)	0.93 (0.92‒0.93)	not performed*
Post-CPB	0.798	0.82 (0.81‒0.83)	*not performed*	0.805

Data are presented as correlation or interclass correlation coefficients (95% confidence interval)

*Abbreviations*: *ICC* Interclass correlation, *T*_*ZHF*_ Temperatures measured by ZHF skin temperature sensor, *T*_*NP*_ Temperatures measured by nasopharyngeal thermometer, *T*_*PAC*_ Temperature measured by PA catheter

^*^PA catheter was withdrawn during the CPB

## Discussion

In this prospective observational study of patients undergoing cardiac surgery with CPB, T_ZHF_ closely tracked nasopharyngeal temperature throughout the intraoperative period. At the primary endpoint, 30 min after CPB weaning, two measurements showed no statistically significant difference, and their mean difference remained small (− 0.12 °C). Across all intraoperative phases, T_ZHF_ and T_NP_ showed consistently high correlations and strong intraclass correlation coefficients (ICC3 ranging from 0.82 to 0.99), indicating that both measurements followed similar temperature trajectories. However, Bland–Altman analysis revealed that the LOA widened during phases involving rapid temperature changes, particularly during CPB cooling and rewarming. These findings suggest that while T_ZHF_ closely reflects the overall temperature trends observed with T_NP_, the gap between the two measurements may widen during rapid and active temperature modulation. From a clinical standpoint, however, temperature monitoring during CPB is often used to guide directional trends and detect phases of cooling or rewarming rather than to rely on absolute values at a single time point, suggesting that tracking performance may be more relevant to clinical decision-making than strict numerical agreement alone.

The agreement observed between T_ZHF_ and T_NP_ in our study was consistent with prior studies demonstrating that T_ZHF_ provides reliable estimates of core temperature under relatively stable thermal conditions, particularly in normothermic and mildly hypothermic temperature ranges (> 32 ℃) during cardiac surgery [13–15]. The mean bias between T_ZHF_ and T_NP_ remained small throughout the intraoperative phases, suggesting that the two monitoring modalities provide comparable temperature estimates at the population level. Nevertheless, despite acceptable agreement and high correlation between T_ZHF_ and T_NP_ measurements (Table 3), their LOA ranged up to ± 2.3 °C during these phases (Fig. 3B). The LOA exceeding the predefined clinical margin (> 0.5 °C) indicated that the two methods are not perfectly interchangeable under dynamic temperature changes during CPB. However, importantly, such discrepancies are not unique to T_ZHF_ monitoring. Similar discrepancies are possible with tissue-based temperature monitoring of nasopharyngeal (T_NP_), esophageal, and bladder temperature measurements, particularly when systemic temperature changes rapidly during CPB. Even T_NP_ would lag behind blood temperature at the CPB outlet and T_PAC_, although it closely tracked changes in CPB blood temperature; this lag is required to compensate for the temperature gradient between NP tissue and blood at the CPB arterial outlet. Especially during CPB cooling and rewarming, T_NP_ lagged behind changes in blood temperature and became a less accurate surrogate for brain temperature due to an additional lag between the blood and brain [7]. Furthermore, for monitoring brain temperature, a larger temperature gradient during rapid temperature changes would necessitate a much longer time lag and compromise the accuracy of T_ZHF_, as well as that of T_NP_ [6, 7]. Although T_PAC_ reflects blood temperature more accurately than T_ZHF_ and T_NP_, it cannot accurately determine brain temperature, especially during rapid temperature changes [5]. In our study, T_PAC_, which represents the actual blood temperature, was not used because PAC was withdrawn during most of the CPB application. T_ZHF_ has the potential to directly reflect brain tissue temperature 1–2 cm below the forehead sensor in a non-invasive manner [15, 17]. Furthermore, the CPB arterial outlet temperature was titrated by perfusionists, and its gap relative to T_NP_ (approximately 1.0–2.0 °C) was usually much greater than the margin, especially during CPB rewarming. This means that even the CPB outlet temperature cannot replace blood temperature measured at other sites, such as T_PAC_, due to blood mixing during rapid temperature changes.

There were dynamic patterns during rapid temperature changes: during CPB cooling, T_ZHF_ measurements changed more gradually than T_NP_ measurements, and their difference increased in the early phase of cooling and gradually decreased as the temperature approached the target level (nadir). During CPB rewarming, T_ZHF_ and T_NP_ showed minimal differences at the beginning. Thereafter, T_ZHF_ became progressively higher than T_NP_, and the temperature difference increased to approximately 0.5°C by the completion of rewarming. This pattern likely reflects the physiological and technical characteristics of zero-heat-flux thermometry, which estimates deep-tissue temperature by creating an isothermal tunnel between the skin surface and the underlying tissues. Establishing this thermal equilibrium takes extra time, which may delay thermal equilibration between the skin sensor and deeper tissue during rapid systemic temperature changes. As mentioned earlier, this phenomenon likely reflects physiological delays in thermal equilibration rather than limitations inherent to the zero-heat-flux technique.

Our study confirmed that no single temperature-monitoring method can reliably measure both blood and brain temperatures, particularly during cardiac surgery, where rapid temperature fluctuations occur. The LOA exceeding the clinically acceptable margin (± 0.5 °C) between T_ZHF_ and T_PAC_, as well as between T_NP_ and T_PAC_, suggested that neither T_ZHF_ nor T_NP_ accurately reflected blood temperature even during the Pre- and Post-CPB periods. This is why recent guidelines recommend routine monitoring of both blood and tissue temperatures, i.e., combined monitoring of T_NP_ and CPB blood temperature, as routine for cardiac surgery with CPB [8].

Considering the risks and complications associated with placing a T_NP_ thermistor or a PAC, combined monitoring of T_ZHF_ and CPB blood temperature may be a good alternative in cardiac surgery. Furthermore, given the importance of careful temperature management [5], the fully non-invasive nature would be an additional benefit of T_ZHF_ for extending the coverage of continuous temperature monitoring— covering the fully awake state and the postoperative ICU stay [3, 4].

Because temperature monitoring inherently produces repeated time-series measurements, we used mixed-effects analysis to account for intra-individual variability and time-dependent effects. Our mixed-effects model demonstrated considerable variability among patients in the difference between T_ZHF_ and T_NP_ measurements during periods of rapid temperature change (Supplementary Fig. 5), especially due to patient-specific factors, including target hypothermic temperature, cooling and rewarming rates, and local tissue perfusion. Such variability further supports the concept that temperature monitoring during CPB should be interpreted in the context of overall trends rather than relying solely on absolute values from a single monitoring site. Therefore, while Bland–Altman limits of agreement highlight that T_ZHF_ and T_NP_ are not interchangeable as absolute surrogates for blood or brain temperature during dynamic phases, our analyses of tracking ability support the practical use of T_ZHF_ to follow intraoperative temperature trends and to identify clinically important transitions such as onset and completion of hypothermic management [30].

Several limitations were present in our study. T_NP_ was used as the reference method for comparison, although no single temperature monitoring site can be considered a definitive gold standard for core temperature during cardiac surgery. T_PAC_ data were unavailable because the PAC was withdrawn during CPB warming, and blood temperature measurements at the CPB arterial outlet were not included in the present study. The lack of blood temperature data limits comparisons with measurements taken during active temperature modulation. Temperature values below 25 °C were excluded from the analysis due to technical limitations of the T_ZHF_ monitoring system. Although the study included a large number of temperature measurements, the data were derived from a relatively small number of patients, and repeated measurements within individuals may introduce clustering effects. Mixed-effects models were used to account for intra-individual variability; nevertheless, the findings should be interpreted in the context of the study design.

## Conclusion

Our study showed that T_ZHF_ monitoring provides a practical and non-invasive method for tracking intraoperative T_NP_ trends during cardiac surgery with CPB. However, the LOA between the two measurements widened during periods of rapid temperature change, particularly during CPB cooling and rewarming, thereby limiting the accuracy and reliability of T_ZHF_. These findings provide useful insights into the lack of a single modality capable of precisely and reliably monitoring both blood and brain temperatures, and into the dynamic behavior of temperature monitoring, especially during active temperature modulation in cardiac surgery. Taken together, these findings underscore that, for intraoperative temperature management in cardiac surgery, the capacity of a monitoring modality to faithfully track temporal changes and inform trend-based decisions may be more clinically meaningful than achieving narrow limits of absolute agreement with a reference site.

## Supplementary Information

Supplementary Material 1.

## Data Availability

The datasets generated and/or analyzed during the current study are available from the corresponding author on reasonable request.

## Abbreviations

AVR: Aortic valve replacement
CI: Confidence interval
CPB: Cardiopulmonary bypass
CPB-cooling: Phase during the CPB cooling
CPB-maintenance: Phase during the maintenance of hypothermic CPB (> 32 ℃)
CPB-warming: Phase during the CPB rewarming
ICC: Interclass correlation
LOA: Limit of agreement
MVP: Mitral valve plasty
MVR: Mitral valve replacement
NP: Nasopharynx
PAC: Pulmonary artery catheter
Pre-CPB: Phase before applying CPB
Post-CPB: Phase after weaning from CPB
SD: Standard deviation
TAP: Tricuspid annuloplasty
T_NP_: Temperatures measured by nasopharyngeal thermometer
T_PAC_: Temperature measured by PA catheter
T_ZHF_: Temperatures measured by ZHF skin temperature sensor
ZHF: Zero-heat-flux technique
