# Supplementary material for: Zero-heat flux skin temperature tracks nasopharyngeal temperature during cardiac surgery with cardiopulmonary bypass: a prospective observational study
Source: BMC Anesthesiol. 2026 Jul 14;26:612. doi: 10.1186/s12871-026-04068-4 (PMC13660041; doi:10.1186/s12871-026-04068-4)
Supplement: Supplementary file 1 [file 12871_2026_4068_MOESM1_ESM.docx]

**Supplementary Table 1. Bland-Altman analysis between T_ZHF_, T_NP_, and T_PAC_ measurements**

|  |  | Mean difference, bias (95% CI) (℃) | SD of differences (℃) | Upper LOA  (95% CI) (℃) | Lower LOA  (95% CI) (℃) | Proportion  outside LOA (%) |
| --- | --- | --- | --- | --- | --- | --- |
| T_ZHF_ vs T_NP_ | Pre-CPB | 0.073  (0.054 ‒ 0.092) | 0.376 | 0.811  (0.779 ‒ 0.843) | -0.664  (-0.697 ‒ -0.632) | 6.70 |
|  | CPB-cooling | 0.281  (0.243 ‒ 0.319) | 0.679 | 1.612  (1.548 ‒ 1.677) | -1.050  (-1.115 ‒ -0.986) | 5.05 |
|  | CPB-maintenance | -0.209  (-0.243 ‒ -0.175) | 0.459 | 0.690  (0.632 ‒ 0.749) | -1.109  (-1.167 ‒ -1.050) | 0.86 |
|  | CPB-rewarming | -0.550  (-0.596 ‒ -0.504) | 0.897 | 1.207  (1.130 ‒ 1.285) | -2.307  (-2.385 ‒ -2.230) | 6.21 |
|  | Post-CPB | -0.181  (-0.196 ‒ -0.165) | 0.465 | 0.730  (0.704 ‒ 0.756) | -1.092  (-1.118 ‒ -1.066) | 4.45 |
| T_ZHF_ vs T_PAC_ | Pre-CPB | -0.047  (-0.065 ‒ 0.030) | 0.341 | 0.622  (0.592 ‒ 0.652) | -0.716  (-0.746 ‒ -0.687) | 5.85 |
|  | CPB-cooling* | - | - | - | - | - |
|  | CPB-maintenance* | - | - | - | - | - |
|  | CPB-rewarming* | - | - | - | - | - |
|  | Post-CPB | -0.373  (-0.389 ‒ -0.357) | 0.461 | 0.530  (0.503 ‒ 0.557) | -1.277  (-1.303 ‒ -1.250) | 4.15 |
| T_NP_ vs T_PAC_ | Pre-CPB | -0.149  (-0.162 ‒ -0.136) | 0.250 | 0.341  (0.319 ‒ 0.363) | -0.640  (-0.662 ‒ -0.618) | 4.46 |
|  | CPB-cooling* | - | - | - | - | - |
|  | CPB-maintenance* | - | - | - | - | - |
|  | CPB-rewarming* | - | - | - | - | - |
|  | Post-CPB | -0.19  (-0.201 ‒ -0.178) | 0.323 | 0.444  (0.425 ‒ 0.463) | -0.823  (-0.842 ‒ -0.804) | 6.70 |

**Abbreviations:** T_ZHF_ = temperatures measured by ZHF skin temperature sensor, T_NP_ = temperatures measured by nasopharyngeal thermometer, T_PAC_ = temperature measured by PA catheter, 95% CI = 95% confidence interval, LOA = limit of agreement, SD = standard deviation.

* = T_PAC_ data during CPB-cooling, CPB-maintenance, and CPB-rewarming were excluded from the analyses.

**Supplementary Table 2. Mixed effect models for T_ZHF_ and T_NP_ changes during the progress of CPB cooling and CPB warming**

|  | | Progress of CPB-cooling | | | |  | | Progress of CPB-rewarming | |
| --- | --- | --- | --- | --- | --- | --- | --- | --- | --- |
| **Fixed effect** | | | Estimate (95% CI) | | P value |  | | Estimate (95% CI) | P value |
|  | Intercept | | -0.135  (-0.295 ‒ 0.0255) | | 0.103 |  | | 0.393  (0.186 ‒ 0.600) | <0.001 |
|  | Cooling/rewarming progress | | -0.001  (-0.003 ‒ 0.001) | | 0.204 |  | | 0.002  (-0.001 ‒ 0.005) | 0.233 |
| **Random effect** | | | SD | | correlation coefficient |  | | SD | correlation coefficient |
|  | Individual intercept | | 0.440 | | - |  | | 0.571 | - |
|  | Individual cooling/rewarming progress | | 0.005 | | - |  | | 0.008 | - |
|  | Correlation between intercept and slope | | - | | -0.39 |  | | - | -0.10 |
| **Model summary** | |  | | | | | | | |
|  | Akaike Information Criterion | | | 1119.9 | |  | 1168.1 | | |
|  | Bayesian Information Criterion | | | 1150.6 | |  | 1199.9 | | |
|  | Comparison with the random intercept-only model (P value) | | | <0.001 | |  | <0.001 | | |

**Abbreviations:** T_ZHF_ = temperatures measured by ZHF skin temperature sensor, T_NP_ = temperatures measured by nasopharyngeal thermometer, 95% CI = 95% confidence interval, SD = standard deviation,

**Supplementary Figure 1. Bland-Altman plot of T_ZHF_ and T_NP_ measurements during five different intraoperative phases.**

A = Pre-CPB,B = CPB-cooling, C = CPB-maintenance, D = CPB-rewarming, E = Post-CPB.

**Abbreviations:** T_ZHF_ = temperatures measured by ZHF skin temperature sensor, T_NP_ = temperatures measured by nasopharyngeal thermometer.

**Supplementary Figure 2. Bland-Altman plot of T_ZHF_ and T_PAC_ measurements during CPB cooling and CPB rewarming phases.**

A = Pre-CPB, B = Post-CPB.

**Abbreviations:** T_ZHF_ = temperatures measured by ZHF skin temperature sensor, T_PAC_ = temperature measured by PA catheter.

**Supplementary Figure 3. Bland-Altman plot of T_NP_ and T_PAC_ measurements during CPB cooling and CPB rewarming phases**

A = Pre-CPB, B = Post-CPB.

**Abbreviations:** T_NP_ = temperatures measured by nasopharyngeal thermometer, T_PAC_ = temperature measured by PA catheter.

**Supplementary Figure 4. The difference between T_ZHF_ and T_NP_ measurements throughout the normalized active temperature modulation during CPB cooling and rewarming phases**

A = CPB-cooling phase, B = CPB-rewarming phase.

**Abbreviations:** T_ZHF_ = temperatures measured by ZHF skin temperature sensor, T_NP_ = temperatures measured by nasopharyngeal thermometer.

**Supplementary Figure 5. Changes in temperature difference between T_NP_ and T_ZHF_ during CPB cooling and rewarming phases**

A = CPB cooling phase, B = CPB rewarming phase. The black lines indicate population changes, and the grey dotted lines indicate individual temperature changes during the progress of CPB-cooling and CPB-rewarming procedures. The progress of active temperature change is normalized according to the duration of CPB-cooling and CPB-rewarming phases.

**Abbreviations:** T_ZHF_ = temperatures measured by ZHF skin temperature sensor, T_NP_ = temperatures measured by nasopharyngeal thermometer.

s
